# How many photons are needed for FRET imaging?

**DOI:** 10.1364/BOE.379305

**Published:** 2020-01-30

**Authors:** Alessandro Esposito

**Affiliations:** MRC Cancer Unit, University of Cambridge, Biomedical Campus, Cambridge, CB20XY, UK

## Abstract

Förster resonance energy transfer (FRET) imaging is an essential analytical method in biomedical research. The limited photon-budget experimentally available, however, imposes compromises between spatiotemporal and biochemical resolutions, photodamage and phototoxicity. The study of photon-statistics in biochemical imaging is thus important in guiding the efficient design of instrumentation and assays. Here, we show a comparative analysis of photon-statistics in FRET imaging demonstrating how the precision of FRET imaging varies vastly with imaging parameters. Therefore, we provide analytical and numerical tools for assay optimization. Fluorescence lifetime imaging microscopy (FLIM) is a very robust technique with excellent photon-efficiencies. However, we show that also intensity-based FRET imaging can reach high precision by utilizing information from both donor and acceptor fluorophores.

## 1. Introduction

Förster resonance energy transfer (FRET) is the non-radiative transfer of energy from a donor fluorophore to an acceptor chromophore [1,2]. The probability for a molecule to transfer energy via FRET (*E*, FRET efficiency) is typically sensitive to distances within the nanometers range [3]. Therefore, for its high sensitivity at the nanometer scale, FRET has many applications in biophysics and biomedical sciences (reviewed in [4–7]). FRET results in the reduction of the quantum yield and the fluorescence lifetime of the donor fluorophore. In the instances where the acceptor is fluorescent, FRET also causes acceptor sensitized emission [1]. The quantification of the intensity emitted by an acceptor normalized to the signal emitted by a donor fluorophore is often referred as sensitized emission FRET or seFRET. Fluorescence lifetime imaging microscopy (FLIM) [8–10] is one of the methodologies that enables researcher to quantitate FRET; among the various implementations of FLIM [1,5,11], time-correlated single-photon counting (TCSPC) is regarded as the gold-standard for its high precision and accuracy [12,13].

**Fig. 1.**
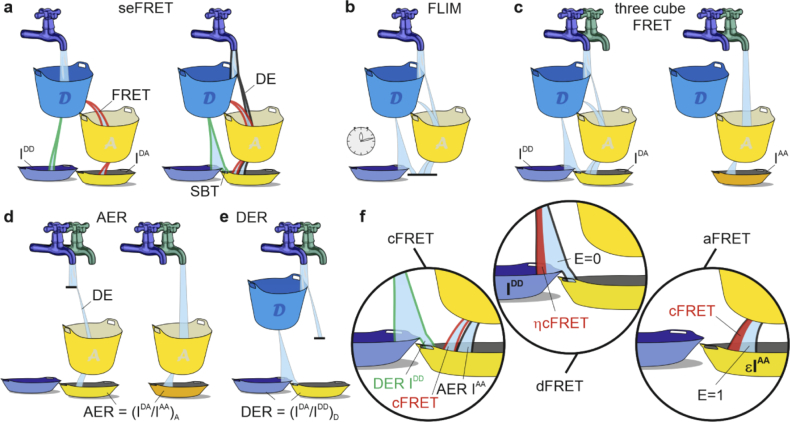
Quantification of FRET by FLIM and seFRET. Excitation light pumps a population of excited fluorophores, here represented as blue (donor) or yellow (acceptor) buckets. In an ideal system, the light source (blue tap) excites only the pool of donor fluorophores directly (a). Excitation energy then is either transferred non-radiatively to the acceptor or emitted as photons and collected by detectors, here represented by the bottom blue (I^DD^) and yellow (I^DA^) plates. The ratio of I^DA^ and I^DD^ can be used to estimate FRET. In practice, direct excitation of the acceptor (DE) and spectral bleed-through from donor to acceptor (SBT) contaminate the FRET signal. FLIM (b) avoids cross-talks between donor and acceptor by estimating the presence of FRET by the time a fluorophore spends in its excited state. Quantitative implementations of seFRET requires the estimation of cross-talks using a third image (I^AA^ in c) from which correction factors (AER in d, and DER in e) can be estimated. These parameters are used to subtract spill-over contributions from the FRET-sensitized acceptor emission (f, cFRET). FRET efficiency (E) can be then estimated by normalizing cFRET to the donor (dFRET) or acceptor emission (aFRET) that would have been measured with E = 0 or E = 1, respectively.

Figure 1 illustrates concepts that are useful to understand FRET detection by comparing the flow of information from light source, fluorophores to detectors with an analogy to the flow of a liquid. Excitation light pumps the exited state of a fluorophore (Fig. 1(a) which then decays to its ground state emitting light, as if it was water (green shaded) dripping from a hole at the bottom of a bucket into another container (the detector). FRET provides a second de-excitation pathway that permits energy to flow to an acceptor that will dissipate its energy by emitting red-shifted photons. Measurements of donor (I^DD^) and acceptor (I^DA^) intensities excited at a wavelength optimized for donor excitation provide a quantification of FRET. In practice, excitation light directly pumps the excited state of an acceptor fluorophore (Fig. 1(a); direct excitation, DE), and spectral emission overlap between fluorophores causes light from the donor to ‘spill-over’ into the acceptor channel (Fig. 1(a); spectral bleed-through, SBT). Cross-talks thus render ratiometric FRET sensitive to the relative concentration of donor and acceptor fluorophores. Rather than measuring the relative intensities of a FRET pair, FLIM quantifies FRET by measuring the average time that donor molecules spend in the excited state (Fig. 1(b), thus avoiding the need to correct for cross-talks. Intensity-based techniques, however, require corrections that are provided by multi-colour (referred to as three -channel or -cube, corrected or precision FRET [14–18]) or hyperspectral imaging [19–22].

Quantitative seFRET requires at least the acquisition of the acceptor fluorescence excited at a wavelength optimized for the excitation of the acceptor (I^AA^, Fig. 1(c). The acceptor excitation ratio AER = [I^DA^/I^AA^]_A_ – obtained by exciting a sample containing only the acceptor fluorophores (Fig. 1(d) – enables the estimation of direct excitation. Similarly, the donor emission ratio DER = [I^DA^/I^DD^]_D_ - measured with a sample containing only the donor fluorophore - is used to estimate the donor spectral bleed-through (Fig. 1(e). The corrected FRET signal (*cFRET*) can be then evaluated for each pixel as: *cFRET = I^DA^-DER I^DD^-AER I^AA^* (Fig. 1(f) and [14,15]). FRET efficiency is estimated by relating cFRET to the intensity that would have been emitted by the donor *dFRET =η cFRET / (I^DD^+ η cFRET)* or acceptor *aFRET =ɛ cFRET / I^AA^* either if FRET did not occur or if E = 100%, respectively (Fig. 1(f). ɛ and η are the ratio of the donor/acceptor excitation light intensities and detection efficiencies, respectively, parameters that are measured with a reference sample of known FRET efficiency (see Appendix 2). dFRET and aFRET are good estimators for the apparent FRET efficiency [14], *i.e.*, E multiplied by the fraction of interacting donors (f_D_) or acceptors (f_A_), respectively. Protocols for the estimation of seFRET are described in [14,15,23] and comparison between different nomenclatures are shown in Tables 3–5.

Biological applications of FRET and FLIM are constrained by the limited photon-budget available, *i.e.* the number of detectable photons within a reasonable exposure time limited by photodamage and phototoxicity, or by the spatiotemporal and biochemical resolutions required to characterize dynamic biological processes. The role of photon-statistics in FRET imaging has been characterized, more extensively for FLIM applications [8,10,24–31] and, to our knowledge, at a lesser extent for intensity-based techniques [32,33]. Here, we study the role of photon-statistics in seFRET and provide a theoretical comparison of the physical limits in precision between seFRET and TCSPC. Interestingly, seFRET performs very well from a theoretical perspective, resulting in high precision because of the efficient utilization of information from both donor and acceptor signals, suggesting strategies to enhance the biochemical resolving power in FRET microscopy.

## 2. Results

### 2.1 Fisher information matrix and seFRET

**How many photons are necessary to estimate FRET?** The answer to this question depends on the precision we want to achieve. In addition to other sources of errors not considered in this work, fluorescence detection always exhibits at least Poissonian noise [25,32,34]. Fisher information theory permits us to estimate the Cramer-Rao lower bound (σ, CRLB), *i.e.* the smallest achievable statistical error in the estimate of a random variable. Given N_D_ detected photons, we can often write $\sigma =\tilde{\sigma }N_{D}^{-0.5}$ where $\tilde{\sigma }$ depends on imaging parameters but not on N_D_. Therefore, $\tilde{\sigma }$ represents the photon-efficiency of a method. To compute $\tilde{\sigma }$, we first developed the analytical description of the Fisher information for a three-filter seFRET that is used for live cell imaging [14,15,17,35] following the method described in the seminal work of Watkins *et al.* originally for single-molecule FRET (Appendix 1 and Ref. [32]). In single-molecule detection, I^AA^ can be disregarded, but its measurement has significant implications for seFRET. The step-by-step analytical derivation of our analytical framework is described in Appendix 2. Briefly, we evaluated the Fisher information matrix *J* and the element (J^−1^)_11_ of its inverse that gives CRLB [32,36] for the variance of dFRET (i = D) and aFRET (i = A):

(1) $$\sigma_{E_{i}}^{2}={\left({J_{iFRET}}^{-1}\right)}_{11}=N_{P}^{-1}({\tilde{\sigma }}_{B}^{2}+{\tilde{\sigma }}_{SBT}^{2}+{\tilde{\sigma }}_{E}^{2})$$

${\tilde{\sigma }}_{E_{i}}^{2}={\tilde{\sigma }}_{B}^{2}+{\tilde{\sigma }}_{SBT}^{2}+{\tilde{\sigma }}_{E}^{2}$ describes the contribution of background, spectral bleed-though and FRET efficiency to the standard deviation of the FRET estimators (see Eqs. (32)–(34) for dFRET and Eqs. (35)–(37) for aFRET in Appendix 2 for analytical descriptions).

**Fig. 2.**
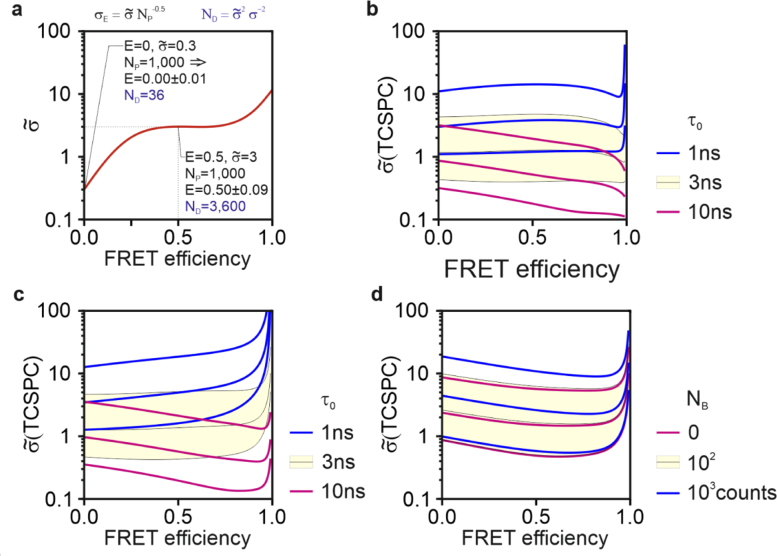
Photon-economy in FRET estimation by TCSPC. The $\tilde{\sigma }$ values were obtained numerically. The mock curve in (a) exemplifies how the $\tilde{\sigma }$ values can be used. Divided by N_P_^0.5^, $\tilde{\sigma }$ returns the expected standard deviation on the FRET estimate. When squared and divided by the maximum variance that might be targeted in an experiment, $\tilde{\sigma }$ provide an estimate of the minimum number of photons that should be collected (N_D_). Numerical estimations of the standard deviations of FRET estimates measured with FLIM, for an ideal system (b) with Dirac-like IRF for *τ*_0_=1ns (blue), 3ns (black lines and yellow area) and 10ns (magenta) or with a finite IRF of 38ps fwhm (c). Curves of the same color show f = 10%, 50% and ∼90% from top to bottom. (d) Simulations for *τ*_0_=3ns, with an uncorrelated background that must be estimated, with values of 0 (magenta), 100 (black curves and yellow area) and 1,000 photons (blue).

**Fig. 3.**
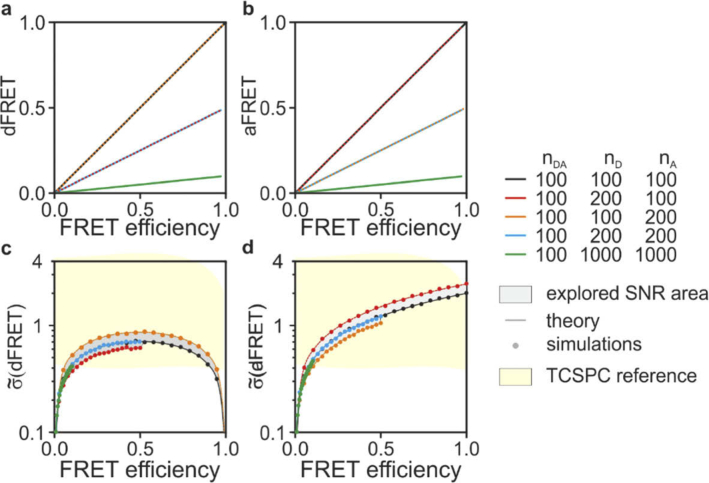
seFRET in the absence of background. The dFRET (a) and the aFRET (b) estimators are unbiased in the absence of background signals and, as expected they estimate the quantities f_D_E and f_A_E, respectively. The intensity-normalized standard deviations for dFRET (c) and aFRET (d) vary with a sweep of the parameters (n_DA_, n_D_, n_A_ and E) albeit in a narrow SNR area (gray) and with a perfect match between the analytical solutions (dark gray curves) and the numerical simulations (circles). In yellow, the reference area explored by TCSPC from Fig. 2(b) is shown.

**Fig. 4.**
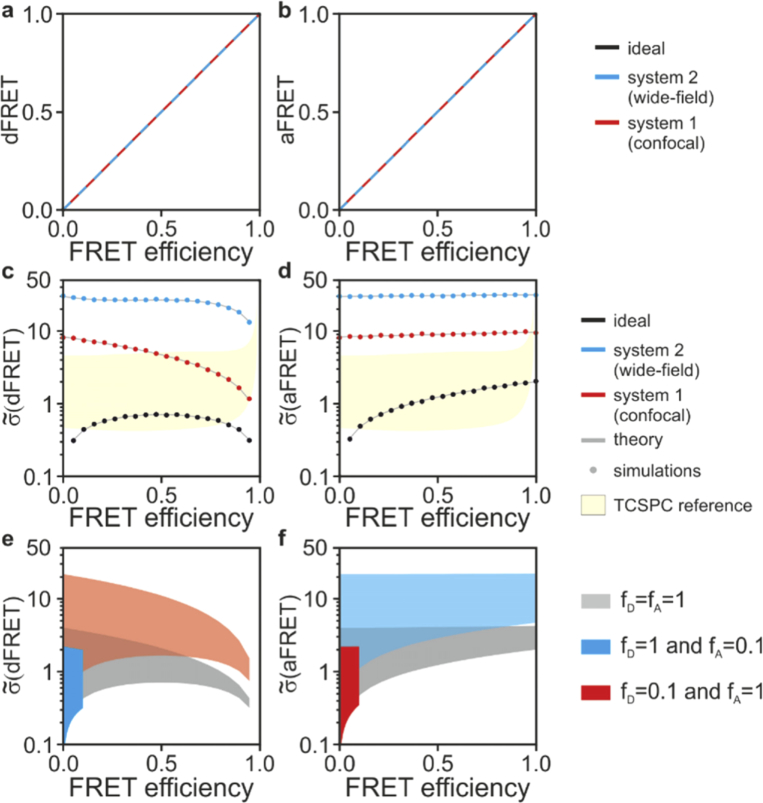
seFRET in the presence of spectral bleed-through. dFRET (a) and aFRET (b) are unbiased estimators as shown using the cross-talk reported in Table 1 for a representative configuration of a confocal (system 1, red) and a wide-field (system 2, blue) microscope. Crosstalk causes a significant deterioration of SNR values for dFRET (c,e) and aFRET estimators (d,f). The loss of SNR is shown in (c-d) and its dependency on DER, AER and the fraction of interacting donor/acceptor fluorophores is further illustrated in (e-f) where the SNR regions for f_D_=f_A_=1 (grey), f_D_=0.1 and f_A_=1 (red) or f_D_=1 and f_A_=0.1 (blue) are shown by varying DER and AER from 0 to 1. In yellow, we show also the TCSPC reference area from Fig. 2(c).

**Fig. 5.**
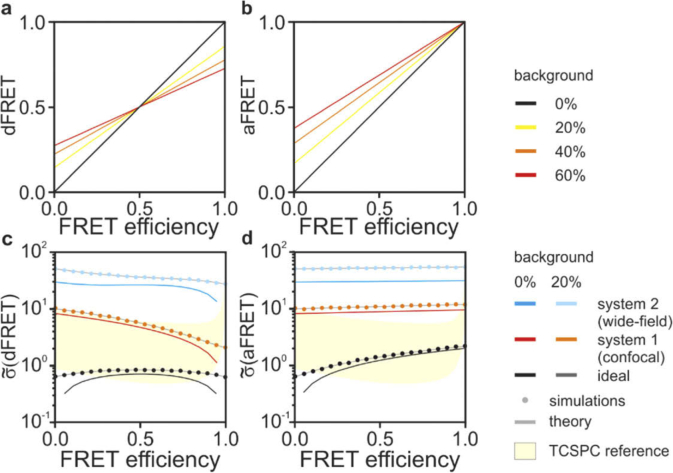
seFRET in the presence of background. The dFRET (a) and aFRET (b) are not accurate estimators of f_D_E and f_A_E in the presence of background (simulated background-to-signal ratio of 0%, black; 20%, yellow; 40%, orange and 60%, red). The analytical solutions (solid lines) describing the noise in dFRET (c) and aFRET (d) match the numerical simulations (solid circles) also in the presence of a background signal. We compare the noise for the systems also shown in Fig. 3, *i.e.* system 1 (confocal, 0% (red) and 20% (orange) background), system 2 (wide-field, 0% (blue) and 20% (cyan) background) and the reference ideal case (0% (black) and 20% (dark gray) background). The SNR range explored by TCSPC from Fig. 2(d) is shown in yellow.

Therefore, ${\tilde{\sigma }}_{E_{i}}^{}$ is a representation of how statistical errors for the FRET estimators scale relative to N_P_. In the next sections, we describe ${\tilde{\sigma }}_{E_{i}}^{}$-curves as a function of FRET efficiency and experimental conditions. Figure 2(a) provides guidance to interpret Figs. 2–5. For example, we can estimate that at E = 0, $\tilde{\sigma }$=0.3 and with N_P_=1,000 photons we would then expect to measure E∼0.00 ± 0.01 ($\sigma =\tilde{\sigma }N_{P}^{-1/2}$). Conversely, $\tilde{\sigma }$-values can also permit us to estimate the number of photons ($N_{D}={\tilde{\sigma }}^{2}\sigma^{-2}$, see Eq. (38) needed to attain a predefined statistical error. For instance, if we set σ = 0.05, from the curve shown in Fig. 2(a) we infer that a budget of 3,600 photons is necessary ($N_{D}={\tilde{\sigma }}^{2}\sigma^{-2}$) to estimate E∼0.50 ± 0.05 (see Table 1 for a few case studies).

**Table 1.** Examples of photon-budget required to attain a standard deviation of 5% in FRET efficiency.

case study		TCSPC			dFRET			aFRET		

E	f_D_	$\tilde{\sigma }$	N_D_^*a*^	E	$\tilde{\sigma }$ ^*b*^	N_D_	E	$\tilde{\sigma }$ ^*b*^	N_D_	E
50	50	1.4	1,150(1,500)	50	0.53/3.5	110/5,000	25	1.5/9.4	900/35,000	50
20	20	2.1	1,800(1,900)	20	0.21/2	20/1,600	4	1.5/10.5	900/42,000	20
75	98	0.5	100(370)	75	0.6/2.6	150/2,700	74	1.6/9.3	1,000/35,000	75
75	36	10	40,000(51,000)	75	0.52/2.7	110/2,900	27	2.2/10	1,900/41,500	75
20	98	0.43	75(90)	20	0.56/6.7	190/18,000	20	0.7/8.6	200/30,000	20

^*a*^ Number of photons required for donor imaging by FLIM (available photon-budget including SE)

^*b*^ values of $\tilde{\sigma }$ in the absence of cross-talks (as in Fig. 3) / values in the presence of cross-talks (as in Fig. 4, confocal system)

### 2.2 Cramer-Rao lower bound for TCSPC

To provide a reference for the theoretical efficiency of seFRET, we studied the expected statistical error for the estimation of FRET by TCSPC, the gold-standard in FLIM detection [5]. For the estimation of FRET, we consider a double-exponential model with a known unquenched fluorescence lifetime (*τ*_0_) and total photon counts (N_P_), and with unknown fractional contribution (*f*) and FRET-dependent lifetime (*τ*_0_(1-E) to be fitted. We could not calculate the analytical solutions for this model. Therefore, we studied the problem numerically (see Methods) by adapting code originally developed by Bouchet *et al.* [36]. The Cramer-Rao lower bound for the standard deviation of the FRET estimate is shown in Fig. 2(b)–(d). In Fig. 2(b) we assumed an ideal Dirac-like instrument response function (IRF), for *τ*_0_=1, 3 and 10 ns. For each of these values, we varied *f* from 10% (higher curves), 50% (middle curves) and ∼90% (lower curves) maintaining the number of photons emitted by non-interacting donors at 1,000 and varying the number of photons emitted by the interacting donor from 100, 500 to 10,000. As expected, for larger values of *τ*_0_ and *f*, the normalized standard deviation is lower. Figure 2(c) shows the same analysis but with a finite IRF of ∼38ps full-width at half-maximum (fwhm) as defined in [36]. The IRF has a significant impact only for high FRET efficiencies values when the fluorescence lifetime estimates are in the order of magnitude of the IRF. In Fig. 2(d), we kept *τ*_0_ constant (3ns) but varied the contribution of uncorrelated background from 0 (Fig. 1(c), bottom curves) to 100 (middle) and 1,000 (top) photons. The signal-to-background ratio (SBR) is 1, 5 and 100 (100 photons) and 0.1, 0.5 and 1 (1,000 photons) for f = 10, 50, 90%, respectively. The statistical error in FRET estimates are comparatively robust to the presence of background. We remark that although here we report the number of photons used for the numerical simulations, $\tilde{\sigma }$ does not depend on the specific photon counts we simulated but only on their fractional contribution to a FRET-dependent signal (*e.g.*, *f* or *SBR*).

### 2.3 Photon-economy of seFRET in the absence of cross-talks

First we consider the case where only intrinsic noise is present with η and ɛ set to one to aid the interpretation of the results. Figure 3 shows numerical simulations (see Methods for details) carried out with one-hundred donor-acceptor pairs participating (E from 0% to 99%) in the presence and absence of donor and acceptor molecules that do not undergo energy transfer (f_D_=10-100%, f_A_=10-100%). Figure 3(a)–(b) shows that dFRET and aFRET are unbiased estimators for f_D_E and f_A_E, respectively. Figure 3(c)–(d) shows that the signal-to-noise ratio (SNR) in dFRET is always equal or better than aFRET. In these ideal conditions, dFRET is infinitely precise both with no or 100% energy transfer as the absence of signal from either the donor or acceptor channels unequivocally inform about the occurrence of these cases. The SNR values for dFRET and aFRET depend on the relative number of acceptors and donors in the sample; however, the estimators are quite robust in the absence of spurious signals. Indeed, seFRET explores a relatively narrow SNR area when varying the values of f_D_ and f_A_ (Fig. 3(c)–(d), grey area). In comparison, the SNR for an ideal TCSPC (Fig. 2(b) and Fig. 3(c)–(d), yellow area) explores a much wider range at varying contribution of donors interacting with acceptor fluorophores.

### 2.4 Photon-economy of seFRET in the presence of crosstalk

Next, we introduce spectral cross-talks (*i.e.*, non-negligible AER and DER values) to evaluate at which extent these non-idealities degrade the efficiency of seFRET. Table 2 shows crosstalk values that are reported in the literature for a confocal and a wide-field microscope using typical yellow and cyan fluorescent proteins [14,15]. Figure 4(a)–(b) shows that dFRET and aFRET are unbiased estimators for f_D_E and f_A_E also in the presence of crosstalk. However, the noise performance of the estimators (Fig. 4(c)–(d) are significantly deteriorated, resulting into a 20-fold (system 2, blue) and a 5-fold (system 1, red) increase of standard deviations compared to ideal measurements (black). To generalize these results, in Fig. 4(e)–(f) we show noise with a parameter sweep, where we varied the AER and DER values from 0 to 1 with *η* and *ɛ* set to 1. We also simulated three conditions where: all molecules participate to FRET (f_D_=f_A_=1, grey) and only a minority of donor (f_D_=0.1, f_A_=1, red) or acceptor (f_D_=1, f_A_=0.1, blue) molecules contribute to FRET. Lower values of f_D_ and f_A_ causes a significant deterioration of the SNR. Donor imaging by FLIM does not suffer from spectral bleed-through and it is rather robust also to non-idealities such as broadening of the IRF (Fig. 4(c)–(d), yellow areas). In realistic conditions, FRET estimates by TCSPC tend to outperform seFRET methods.

**Table 2.** properties of FRET pairs relevant to seFRET

FRET pair	Microscope	AER	DER	Η	*ɛ*	Reference
CFP-YFP	Confocal (system 1)	0.60	0.42	0.52	6.3	[14]
CFP-citrine	Wide-field (system 2)	0.29	1.07	0.015	42	[15]

### 2.5 seFRET in the presence of a background signal

We also studied how an unspecific background signal deteriorates the performances of dFRET and aFRET. For simplicity, the numerical simulations are carried out assuming an equal relative background in each channel (B^DD^, B^AA^ and B^DA^), set to fractions ranging from 0 to 60%. Figure 5(a)–(b) shows that both dFRET and aFRET are biased and provide inaccurate estimations for FRET efficiency in the presence of background. These inaccuracies can be ameliorated by experimental corrections and, whenever possible, by operating in high SBR conditions. Moreover, background signals deteriorate SNR values for FRET estimations as shown in Fig. 5(c)–(d) for a SBR set to 80% (note the logarithmic scale). For comparison, the lower and upper boundaries of the TCSPC range shown in in Fig. 5(c)–(d) (yellow) corresponds to SBR values equal to infinity (no background) and 50%, respectively. Therefore, fluorescence signals from non-specific stains (*e.g.*, autofluorescence) deteriorate estimate obtained by any techniques. However, TCSPC is very robust to uncorrelated noise (*e.g.*, dark current or stray light) as it can readily infer its value with small compromises to its precision and accuracy.

## 3. Discussion

FRET imaging is a powerful method used to probe cell biochemistry. It is thus not surprising that so many FRET-based assays exist, from *in vitro* single-molecule detection [32] to *in vivo* imaging [37,38], including common applications (*e.g.*, qPCR and related hybridization assays [39]) and specialist uses such as the study of protein conformations, interactions and modifications [40]. Among the many FRET imaging techniques [41], FLIM and seFRET are two of the most common quantitative methods. FLIM – TCSPC in particular – is regarded as the most robust technique for FRET estimation [9] as it requires fewer control samples, and provides robust and reproducible measurements. The choice between FLIM and seFRET often depends on the availability of specialist instrumentation (*e.g.*, for TCSPC) or requirements such as fast acquisition speed (typically better for seFRET). However, breakthroughs in FLIM-enabling technologies [42–48] and data analysis [49–51] are reducing the barrier to adoption for FLIM; as the choice between FLIM and seFRET might slowly drift away from technical constraints, we aimed to develop a comparative analysis of their limits from an information theory perspective. Our work provides guidance for the choice and further optimization of these methodologies. The analytical and numerical tools we developed can be used to compute reference values for different seFRET configurations and TCSPC. As the role of photon-statistics in the various implementations of FLIM (TCSPC, time-gating and frequency-domain) has been studied in-depth [10,12,13,25,52–55] we focused on TCSPC as a representative standard to compare seFRET to. Figures 2–5 and the case studies shown in Table 1 provide an assessment of performances for seFRET and TCSPC in ideal conditions and illustrative cases representative of specific fluorophores employed.

Interestingly, seFRET can outperform TCSPC in the ideal conditions of negligible spectral crosstalk. Here, TCSPC can attain higher SNR only when a majority of donor fluorophores are engaged in FRET or otherwise the dFRET estimator performs significantly better. A better photon-efficiency of the dFRET estimator stems from the capability of dFRET to utilize information from photons emitted from both donor and acceptor molecules. However, the higher precision of dFRET is vastly reduced in the presence of realistic levels of spectral crosstalk or background. We did not consider the additional statistical and systematic errors that the reference measurements required by seFRET causes and other sources of noise manifesting in detectors that do not operate in single-photon counting. Therefore, despite the excellent performance of seFRET compared to TCSPC, the latter might generally outperform seFRET in reproducibility, accuracy and precision in practical implementations. It is important to note that the appropriate optimization of imaging parameters for seFRET can make seFRET rather competitive also for its high precision, something that might be often underestimated. For instance, the use of long-Stokes shift acceptor fluorophores for seFRET, not usually implemented to the best of our knowledge, might result in vast improvements in the SNR of this intensity-based technique. We also note that we compared seFRET to TCSPC as an established gold-standard in FRET detection. Although the analysis we provided is representative of the limits on precision imposed by photon-statistics for lifetime determination and thus FRET/FLIM, there are also many other implementations of FLIM that can be successfully used for FRET estimation [1,5,24]. At high count-rates, for instance, TCSPC deteriorates its precision and accuracy because of photon-losses and distortions of the experimental decays caused by pulse pile-up and detector dead-time [12,56,57]. These losses were not accounted in this framework. Time-gating or frequency-domain FLIM, which photon-efficiency has been well characterized previously can provide high photon-budgets and fast acquisition [10,12,29,52,53].

We note, however, that there are instances where FLIM might lose its competitive edge relative to the simpler seFRET technique from a photon-efficiency perspective. Ultimately, one of the most substantial differences between FLIM and seFRET is that FLIM is typically used for the detection of donor fluorescence, permitting researchers to streamline the use of the visible spectrum or to optimize Foster distances with dark acceptors [58,59], avoiding crosstalk and issues related to chromatic aberrations. On the contrary, seFRET uses the complete photon-budget emitted by the FRET pair. For instance, the frequent cases where FRET-based biosensors do not exhibit sufficient dynamic range in FLIM but work when imaged by seFRET, might be caused by conditions in which typical FLIM applications, not detecting acceptor fluorescence, provide poor SNR values (*e.g.*, high FRET efficiencies or low fractional contributions of specific reporter states). The use of dark chromophores as acceptor molecules is a strategy to increase sensitivity of sensors [58–60] or to clear-up the visible spectrum for multiplexed detection of biochemical reactions [60,61]. However, we can speculate that, in those cases where the benefits of a dark chromophore might be irrelevant, the combination of seFRET and TCSPC (*e.g.*, in dual-colour or hyperspectral FLIM [34,46,62,63]) will provide significant improvements in the precision of FRET estimation. A higher precision leads directly to an improvement in the capability to resolve smaller biochemical differences in living cells. From a theoretical standpoint, this improvement in biochemical resolving power can be understood from the general analysis of Fisher information in multi-dimensional or multi-parametric detection systems (see for example the *photon partitioning theorem* in [34,64]). From a practical point of view, dual-colour fast high-resolution FLIM might be increasingly accessible thanks to the ongoing revolution in time-resolved detection technologies and could provide yet unexplored ideal performances.

## 4. Methods

Analytical solutions were obtained manually, but their consistency was evaluated with the use of Mathematica (Wolfram). Numerical simulations were generated with Matlab (Mathworks) as we shown in Code 1 [65]. The Cramer-Rao lower bound for TCSPC was obtained with parameters sweeps adapting code from [36]. We utilized their methods to compute the standard deviation, normalized to the total (donor) photon counts, of the shorter fluorescence lifetime estimate. This estimate is the fluorescence lifetime quenched via FRET and evaluated from a double-exponential fit with constant background and known IRF. Figure 2(b) was generated using 1,000 photons emitted by non-interacting donor molecules (*i.e.*, fluorophores not participating in FRET) with *τ*_0_=1, 3 or 10ns. Both *τ*_0_ and N_P_ were used as fixed parameters. The number of photons emitted by donors interacting with acceptor fluorophores (*i.e.*, FRET-competent molecule) was varied from 100, 500 to 10,000. E was varied from 0 to 100% in 128 steps on a power series. We used TCSPC as a gold-standard reference and, therefore, we utilized parameters of high-end systems with a laser repetition rate of 80MHz and 256 time-bins. Figure 1(b) was generated in the same way, but using the experimental IRF provided in Ref. [36]. For Fig. 1(c), we simulated only *τ*_0_=3ns. All other parameters the same as in Fig. 1(b), we varied the number of photons in an uncorrelated background (as a fit parameter) for Fig. 1(c), including 0, 100 and 1,000 photons. All the results are shown as normalized by the total photon count emitted by the donor. We validated error propagation in the unmixing equations with numerical simulations. First, we synthesized noiseless images using the same mathematical framework; subsequently, we added Poissonian noise and unmixed the images to determine how noise propagates to the FRET estimates aFRET and dFRET. Results are presented as normalized to the total photon counts as the shapes of the curves presented do not depend on this value (not shown). Results reported in this work were obtained with Dell Precision workstation equipped with an Intel Xeon CPU E5-1620v3 and 64GB of RAM and Matlab 2018a.

## Appendix 1. Fisher information

The Fisher information matrix defines the information content of parameter estimators accordingly to stochastic models of the experiments. The Fisher information matrix for fluorescence lifetime sensing has been derived analytically and studied previously [25–27]. The Fisher information matrix for sensitized emission FRET was described, to our knowledge, only for the case of single-molecule detection [32]. The information content of an experiment can be described by the likelihood function (*L*):

(2) L=∏i=1m1Ni![F(iU)−F((i−1)U)]Nie−[F(iU)−F((i−1)U)]

Here, we broadly adhere to the formalism described in Ref. [26] revised with some nomenclature introduced in Ref. [32]. The likelihood function is the product of the probability to detect *N_i_* (*i *= 1, …, *m*) photons in *m* independent channels, photons that are Poisson distributed with a rate defined by the model function *F*:

(3) $$F(\vec{u},\vec{q})=\int_{0}^{\vec{u}}f(\vec{\upsilon },q)d\vec{\upsilon }$$

where *f(u,q)* is the expected average signal as a function of channel parameters *u* and experimental parameters that should be estimated *q*. For instance, for time-resolved detection, *u* will equal *t* and integration will be carried out over time bins. *U* is the bandwidth of the channel (*e.g.*, spectral bandwidth, time-gate width). *f* can be factorized into parameters such as the excitation rate (*k_ex_*), the integration time of the signal *T*, and two functions that depend on the experimental parameters (*ζ(q)*) and on the detection system (*S(u)*):

(4) $$f(\vec{u},\vec{q}\to )=k_{ex}TS(\vec{u})\zeta (\vec{q})$$

With distributions found in spectroscopy, the elements *j_ij_* of the Fisher information matrix can be computed as the negative of the expectation of the second derivative of *f*:

(5) $$j_{ij}=-E{\left[\frac{\partial^{2}lnf(\vec{u},\vec{q})}{\partial q_{i}\partial q_{j}}\right]}_{\vec{u}}$$

When the signal is described by the Poisson statistics [32], Eq. (5) further simplifies to:

(6) $$j_{jk}=T\sum_{i=1}^{m}\frac{k_{i}S_{i}(\vec{u})}{\zeta_{i}(\vec{q})}\frac{\partial \zeta_{i}(\vec{q})}{\partial q_{j}}\frac{\partial \zeta_{i}(\vec{q})}{\partial q_{k}}$$

In the next sections, we will describe the algebraic manipulations of seFRET formalism required to obtain the separation of variables shown in Eq. (4) that will permit the estimation of Eq. (6). The Fisher information matrix is essential when studying the noise performance of an estimator as the Cramer-Rao theorem states that the lower bounds of the variance of the unbiased estimators of *q* are defined by the inverse of the Fisher information matrix.

## Appendix 2. Fisher information matrix and seFRET

The fluorescence emission as a function of wavelength (*λ*) for the case of seFRET can be described as the sum of photons emitted by the donor fluorophore, photons emitted by sensitized acceptors (SE) and photons emitted by acceptors upon direct excitation (DE) with the donor excitation light source:

(7) $$f(\lambda ,E,n_{D},n_{A},n_{DA})=k_{ex}T\{S_{D}(\lambda )[n_{D}+n_{DA}(1-E)]+n_{DA}S_{SE}(\lambda )E+n_{A}S_{DE}(\lambda )\}$$

S_D_, S_SE_ and S_DE_ include spectral characteristics of the fluorophores and detection system, *e.g.*, quantum yields, molar extinction coefficients, spectral overlaps; *n_D_*, *n_A_* and *n_DA_* are the number of non-interacting donor, non-interacting acceptor and interacting donor-acceptor molecules in the sample, respectively. seFRET detected by the three-filter method is carried out with two acquisition channels for donor- and sensitized- emission that will effectively carry information on energy transfer and a reference acquisition channel with direct excitation of the acceptor. The main difference between single molecule seFRET and seFRET imaging is the requirement in the latter to reference the measured FRET signal to the concentration of the acceptor molecules. To compute the Fisher information matrix, we shall integrate Eq. (7):

(8) $$F(\lambda )=k_{ex}T\left\{[n_{D}+n_{DA}(1-E)]\int_{0}^{\lambda }S_{D}(\lambda )d\lambda +n_{DA}E\int_{0}^{\lambda }S_{SE}(\lambda )d\lambda +n_{A}\int_{0}^{\lambda }S_{DE}(\lambda )d\lambda \right\}$$

From Eq. (7), we can estimate the fluorescence intensity collected within a detection channel optimized for donor emission fluorescence of spectral window *[λ_d1_, λ_d2_]*:

(9) $$I^{DD}=F(\lambda_{d2})-F(\lambda_{d1})=k_{ex}TS_{DD}[n_{D}+n_{DA}(1-E)]$$

The cross-talk between the donor channel and acceptor emission is assumed to be negligible:

(10) $$\begin{array}{ccc} \int_{\lambda_{d1}}^{\lambda_{d2}}S_{D}(\lambda )d\lambda =S_{DD}; & \int_{\lambda_{d1}}^{\lambda_{d2}}S_{SE}(\lambda )d\lambda =0; & \int_{\lambda_{d1}}^{\lambda_{d2}}S_{DE}(\lambda )d\lambda =0; \end{array}$$

It is convenient also to model an unspecific background signal (*B_DD_*) and to introduce *f_A_* and *f_D_*, the fractions of interacting donor or acceptors:

(11) $$I^{DD}=k_{ex}T\{S_{DD}[n_{D}+n_{DA}(1-E)]+B_{DD}\}=k_{ex}T\{S_{DD}N_{D}[1-f_{D}E]+B_{DD}\}$$

Where *N_D_* is the total number of donor fluorophores. In a similar way, the intensity collected in the donor channel can be described as a function of *f_A_* and the total number of acceptor fluorophores (*N_A_*). In summary:

(12) $$\begin{array}{l} I^{DD}(f_{D}E)=k_{ex}T[S_{DD}N_{D}(1-f_{D}E)+B_{DD}]\\ I^{DD}(f_{A}E)=k_{ex}T[S_{DD}N_{D}-S_{DD}N_{A}f_{A}E+B_{DD}] \end{array}$$

Similarly, we can evaluate the intensity collected in the sensitized emission channel by integration of Eq. (8) over the spectral range *[λ_a1_, λ_a2_]*:

(13) $$I^{DA}=k_{ex}T\{[n_{D}+n_{DA}(1-E)]S_{DD}DER+[n_{DA}E+(n_{A}+n_{DA})\varepsilon AER]S_{AA}+B_{DA}\}$$

where *B_DA_* is an unspecific background and

(14) $$\begin{array}{ccc} \int_{\lambda_{a1}}^{\lambda_{a2}}S_{D}(\lambda )d\lambda =S_{DD}DER; & \int_{\lambda_{a1}}^{\lambda_{a2}}S_{SE}(\lambda )d\lambda =S_{AA}; & \int_{\lambda_{a1}}^{\lambda_{a2}}S_{DE}(\lambda )d\lambda =\varepsilon S_{AA}AER \end{array}$$

ɛ is a proportionality factor between the intensity of the excitation light used for donor and acceptor excitation. DER = [I_DA_/I_DD_]_only-donor_ is the donor emission ratio, a control measurement for the donor spectral bleed-through into the acceptor channel using a donor-only control sample performed by measuring the proportion of signal in the acceptor channel relative to the donor channel, and estimating the ratio between the intensities detected in the acceptor. AER = [I_DA_/I_AA_]_only-acceptor_ is the acceptor excitation ratio, a control measurement aimed to estimate the direct excitation of acceptors by measuring the proportion of intensities in the acceptor channel with a donor-only sample with excitation light optimal for donor and acceptor excitation, respectively. Equation (13) can be rewritten using the definitions of *f_D_*, *f_A_*, *N_D_* and *N_A_* as:

(15) $$\begin{array}{l} I^{DA}(f_{D}E)=k_{ex}T\{N_{D}(S_{AA}-S_{DD}DER)f_{D}E+S_{AA}N_{A}\varepsilon AER+S_{DD}N_{D}DER+B_{DA}\}\\ I^{DA}(f_{A}E)=k_{ex}T\{N_{A}(S_{AA}-S_{DD}DER)f_{A}E+S_{AA}N_{A}\varepsilon AER+S_{DD}N_{D}DER+B_{DA}\} \end{array}$$

The intensity value measured in the acceptor reference channel (*I_AA_*) does not depend on energy transfer or the number of donor molecules and can be simply described as:

(16) $$I^{AA}=k_{ex}T\varepsilon (S_{AA}N_{A}+B_{AA})$$

We can simplify the description of the Fisher information matrix using the following set of substitutions, *E_D_=f_D_E*, *E_A_=f_A_E*, *C_D_=S_DD_N_DD_*, *C_A_=S_AA_N_AA_*, η=*S_DD_/S_AA_, N_P_=k_ex_T.* In this work, the number of detected photons is considered equal to the number of absorbed photons (i.e. assuming no losses from the optics or fluorophores) without any loss of generality. E_D_ and E_A_ are the apparent FRET efficiencies measured in seFRET by the donor (dFRET) and acceptor (aFRET) normalized estimators; C_D_ and C_A_ are the relative concentrations of donor and acceptor in arbitrary units; *η* is the ratio of the relative brightness of the donor and the acceptor fluorophores. Substituting the set of definition shown in Eq. (12), (15) and (16), we thus obtain:

(17) $$\{\begin{array}{l} \begin{array}{l} I^{DD}(E_{D},C_{D})=N_{P}[C_{D}(1-E_{D})+B_{DD}]\\ I^{DD}(E_{A},C_{D},C_{A})=N_{P}[C_{D}-\eta C_{A}E_{A}+B_{DD}]\\ I^{DA}(E_{D},C_{D},C_{A})=N_{P}[(\eta^{-1}-DER)C_{D}E_{D}+C_{A}\varepsilon AER+C_{D}DER+B_{DA}] \end{array}\\ \begin{array}{l} I^{DA}(E_{A},C_{D},C_{A})=N_{P}[(1-DER\eta )C_{A}E_{A}+C_{A}\varepsilon AER+C_{D}DER+B_{DA}]\\ I^{AA}(C_{A})=N_{P}\varepsilon [C_{A}+B_{AA}] \end{array} \end{array}$$

Following the formalism introduced in [32], we rewrite Eq. (17) with the parametrization:

(18) $$\{\begin{array}{l} \begin{array}{l} I^{DD}(E_{D},C_{D},C_{A})=F^{DD}[(1-\beta_{DD}^{-1})\zeta_{DD}(E_{D},C_{D},C_{A})+\beta_{DD}^{-1}]\\ F^{DD}=N_{P}(1+B_{DD}) \end{array}\\ \begin{array}{l} \beta_{DD}=(1+B_{DD})B_{DD}^{-1}\\ \zeta_{DD}(E_{D},C_{D},C_{A})=C_{D}(1-E_{D}) \end{array} \end{array}$$

(19) $$\{\begin{array}{l} \begin{array}{l} I^{DD}(E_{A},C_{D},C_{A})=F^{DD}[(1-\beta_{DD}^{-1})\zeta_{DD}(E_{A},C_{D},C_{A})+\beta_{DD}^{-1}]\\ F^{DD}=N_{P}(1+B_{DD}) \end{array}\\ \begin{array}{l} \beta_{DD}=(1+B_{DD})B_{DD}^{-1}\\ \zeta_{DD}(E_{A},C_{D},C_{A})=C_{D}-\eta C_{A}E_{A} \end{array} \end{array}$$

(20) $$\{\begin{array}{l} I^{DA}(E_{D},C_{D},C_{A})=F^{DA}[(1-\beta_{DA}^{-1})\zeta_{DA}(E_{D},C_{D},C_{A})+\beta_{DA}^{-1}]\\ F^{DA}=N_{P}(1+B_{DA})\\ \beta_{DA}=(1+B_{DA})B_{DA}^{-1}\\ \zeta_{DA}(E_{D},C_{D},C_{A})=(\eta^{-1}-DER)C_{D}E_{D}+C_{A}\varepsilon AER+C_{D}DER \end{array}$$

(21) $$\{\begin{array}{l} I^{DA}(E_{A},C_{D},C_{A})=F^{DA}[(1-\beta_{DA}^{-1})\zeta_{DA}(E_{A},C_{D},C_{A})+\beta_{DA}^{-1}]\\ F^{DA}=N_{P}(1+B_{DA})\\ \beta_{DA}=(1+B_{DA})B_{DA}^{-1}\\ \zeta_{DA}(E_{A},C_{D},C_{A})=(1-DER\eta )C_{A}E_{A}+C_{A}\varepsilon AER+C_{D}DER \end{array}$$

(22) $$\{\begin{array}{l} I^{AA}(C_{A})=F^{AA}[(1-\beta_{AA}^{-1})\zeta_{AA}(C_{A})+\beta_{AA}^{-1}]\\ F^{AA}=\varepsilon N_{P}(1+B_{AA})\\ \beta_{AA}=(1+B_{AA})B_{AA}^{-1}\\ \zeta_{AA}(C_{A})=C_{A} \end{array}$$

The Fisher information matrix can be now estimated by computing a set of derivatives of the functions *ζ_s_* and substituting in Eq. (6):

(23) $$\left(\begin{array}{ccc} \frac{\partial \zeta_{DD}}{\partial E_{D}} & \frac{\partial \zeta_{DD}}{\partial C_{D}} & \frac{\partial \zeta_{DD}}{\partial C_{A}}\\ \frac{\partial \zeta_{DA}}{\partial E_{D}} & \frac{\partial \zeta_{DA}}{\partial C_{D}} & \frac{\partial \zeta_{DA}}{\partial C_{A}}\\ \frac{\partial \zeta_{AA}}{\partial E_{D}} & \frac{\partial \zeta_{AA}}{\partial C_{D}} & \frac{\partial \zeta_{AA}}{\partial C_{A}} \end{array}\right)=\left(\begin{array}{ccc} -C_{D} & 1 & 0\\ (\eta^{-1}-DER)C_{D} & DER-(\eta^{-1}-DER)E_{D} & \varepsilon AER\\ 0 & 0 & 1 \end{array}\right)$$

(24) $$\left(\begin{array}{ccc} \frac{\partial \zeta_{DD}}{\partial E_{A}} & \frac{\partial \zeta_{DD}}{\partial C_{D}} & \frac{\partial \zeta_{DD}}{\partial C_{A}}\\ \frac{\partial \zeta_{DA}}{\partial E_{A}} & \frac{\partial \zeta_{DA}}{\partial C_{D}} & \frac{\partial \zeta_{DA}}{\partial C_{A}}\\ \frac{\partial \zeta_{AA}}{\partial E_{A}} & \frac{\partial \zeta_{AA}}{\partial C_{D}} & \frac{\partial \zeta_{AA}}{\partial C_{A}} \end{array}\right)=\left(\begin{array}{ccc} -\eta C_{A} & 1 & -\eta E_{A}\\ (1-DER\eta )C_{A} & DER & \varepsilon AER+(1-DER\eta )E_{A}\\ 0 & 0 & 1 \end{array}\right)$$

Each element of the Fisher information matrix can be then computed accordingly to Eq. (S5:

(25) $$J_{11}=N_{P}C_{D}^{2}\left[\frac{1}{C_{D}(1-E_{D})+B_{DD}}+\frac{\eta^{-1}-DER}{(\eta^{-1}-DER)C_{D}E_{D}+C_{A}\varepsilon AER+C_{D}DER+B_{DA}}\right]$$

(26) $$J_{12}=J_{21}=N_{P}C_{D}\left[-\frac{1}{C_{D}(1-E_{D})+B_{DD}}+\frac{\left(\eta^{-1}-DER)C_{D}[DER-(\eta^{-1}-DER)E_{D}\right]}{(\eta^{-1}-DER)C_{D}E_{D}+C_{A}\varepsilon AER+C_{D}DER+B_{DA}}\right]$$

(27) $$J_{13}=J_{13}=N_{P}C_{D}\frac{(\eta^{-1}-DER)\varepsilon AER}{(\eta^{-1}-DER)C_{D}E_{D}+C_{A}\varepsilon AER+C_{D}DER+B_{DA}}$$

(28) $$J_{22}=N_{P}\left\{\frac{1}{C_{D}(1-E_{D})+B_{DD}}+\frac{{\left[DER-(\eta^{-1}-DER)E_{D}\right]}^{2}}{(\eta^{-1}-DER)C_{D}E_{D}+C_{A}\varepsilon AER+C_{D}DER+B_{DA}}\right\}$$

(29) $$J_{23}=J_{32}=N_{P}\left\{\frac{[DER-(\eta^{-1}-DER)E_{D}]\varepsilon AER}{(\eta^{-1}-DER)C_{D}E_{D}+C_{A}\varepsilon AER+C_{D}DER+B_{DA}}\right\}$$

(30) $$J_{33}=N_{P}{\left(C_{A}+B_{AA}\right)}^{-1}$$

Here, we show explicitly only the evaluation of the Fisher information matrix related to dFRET but similar steps can be used also for aFRET. The Cramer-Rao bound for the variance of *E_D_* is the first element of the inverse matrix with elements described in Eq. (25)–(30).

(31) $$\begin{array}{ll} \sigma_{E}^{2}(dFRET) & =N_{P}^{-1}\{B_{DD}{\left[DER\;\eta \;(1-E_{D})+E_{D}\right]}^{2}C_{D}^{-2}+B_{DA}{\left[\eta \;(1-E_{D})\right]}^{2}C_{D}^{-2}\\ & +B_{AA}{\left[\;AER\;\eta \;(1-E_{D})\right]}^{2}C_{D}^{-2}\varepsilon +AER\;(AER+1)[C_{A}{\left(1-E_{D}\right)}^{2}\eta^{2}]C_{D}^{-2}\varepsilon \\ & +DER[(DER+1)(1-E_{D})\eta +2E_{D}][{\left(1-E_{D}\right)}^{2}\eta ]C_{D}^{-1}\\ & +E_{D}(1-E_{D})[E_{D}(1-\eta )+\eta ]C_{D}^{-1}\} \end{array}$$

The variance in the FRET efficiency estimate is equal to the sum of a background variance (*σ_B_*^2^), a variance which depends on the spectral bleed through (*σ_SBT_*^2^) and a variance which does not depends on background contributions (*σ_E_*^2^) as shown in Eq. (1). We can write simpler analytical solutions for each component:

(32) $$\{\begin{array}{l} \begin{array}{l} {\tilde{\sigma }}_{B}^{2}(dFRET)={\tilde{\sigma }}_{B_{DD}}^{2}(dFRET)+{\tilde{\sigma }}_{B_{DA}}^{2}(dFRET)+{\tilde{\sigma }}_{B_{AA}}^{2}(dFRET)\\ {\tilde{\sigma }}_{B_{DD}}^{2}(dFRET)=B_{DD}{\left[DER\;\eta (1-E_{D})+E_{D}\right]}^{2}C_{D}^{-2} \end{array}\\ \begin{array}{l} {\tilde{\sigma }}_{B_{DA}}^{2}(dFRET)=B_{DA}{\left[\eta (1-E_{D})\right]}^{2}C_{D}^{-2}\\ {\tilde{\sigma }}_{B_{AA}}^{2}(dFRET)=B_{AA}{\left[AER\;\eta (1-E_{D})\right]}^{2}C_{D}^{-2}\varepsilon \end{array} \end{array}$$

(33) $$\{\begin{array}{l} {\tilde{\sigma }}_{SBT}^{2}(dFRET)={\tilde{\sigma }}_{DER}^{2}(dFRET)+{\tilde{\sigma }}_{AER}^{2}(dFRET)\\ {\tilde{\sigma }}_{DER}^{2}(dFRET)=AER(AER+1)[C_{A}{\left(1-E_{D}\right)}^{2}\eta^{2}]C_{D}^{-2}\varepsilon \\ {\tilde{\sigma }}_{AER}^{2}(dFRET)=DER[(DER+1)(1-E_{D})\eta +2E_{D}][{\left(1-E_{D}\right)}^{2}\eta ]C_{D}^{-1} \end{array}$$

(34) $${\tilde{\sigma }}_{E}^{2}(dFRET)=E_{D}(1-E_{D})[E_{D}(1-\eta )+\eta ]C_{D}^{-1}$$

Similarly, we can evaluate the analytical descriptions for the noise of the estimator aFRET:

(35) $$\{\begin{array}{l} {\tilde{\sigma }}_{B}^{2}(aFRET)={\tilde{\sigma }}_{B_{DD}}^{2}(aFRET)+{\tilde{\sigma }}_{B_{DA}}^{2}(aFRET)+{\tilde{\sigma }}_{B_{AA}}^{2}(aFRET)\\ {\tilde{\sigma }}_{B_{DD}}^{2}(aFRET)=B_{DD}DER\;C_{A}^{-2}\\ {\tilde{\sigma }}_{B_{DA}}^{2}(aFRET)=B_{DA}C_{A}^{-2}\\ {\tilde{\sigma }}_{B_{AA}}^{2}(aFRET)=B_{AA}{\left[\varepsilon \;AER\;E_{A}\right]}^{2}C_{D}^{-2}\varepsilon^{-1} \end{array}$$

(36) $$\{\begin{array}{l} {\tilde{\sigma }}_{SBT}^{2}(aFRET)={\tilde{\sigma }}_{DER}^{2}(aFRET)+{\tilde{\sigma }}_{AER}^{2}(aFRET)\\ {\tilde{\sigma }}_{DER}^{2}(aFRET)=DER(DER+1)[C_{D}-C_{A}E_{A}\eta ]C_{A}^{-2}\\ {\tilde{\sigma }}_{AER}^{2}(aFRET)=AER[(AER+1)\varepsilon +2E_{A}]C_{A}^{-1} \end{array}$$

(37) $${\tilde{\sigma }}_{E}^{2}(aFRET)=E_{A}(1+\varepsilon^{-1}A)C_{A}^{-1}$$

A comparison between different nomenclatures used in the literature is shown in Tables 3–5.

**Table 3.** Conversion of nomenclature from Elder et al. [14]

	AER	DER	*α*	*β*
In this work	AER	DER	ηDER	(AERɛ)^−1^
In Hoppe *et al.*	*α*	*β*	*ξβγ*^−1^	*γ*^−1^

**Table 4.** Conversion of nomenclature from this work

	AER	DER	*η*	*Ε*
In Elder *et al.*	AER	DER	α/DER	β/AER
In Hoppe *et al.*	*α*	*β*	*ξγ*^−1^	(*αγ*)^−1^

**Table 5.** Conversion of nomenclature from Hoppe et al. [15]

	Α	*β*	*ξ*	Γ
In this work	AER	DER	*η*(AER*ɛ*)^−1^	AER*ɛ*
In Elder *et al.*	AER	DER	*α*(DER*β*)^−1^	*β*^−1^
